# Quantitation of putative colorectal cancer biomarker candidates in serum extracellular vesicles by targeted proteomics

**DOI:** 10.1038/s41598-017-13092-x

**Published:** 2017-10-06

**Authors:** Takashi Shiromizu, Hideaki Kume, Mimiko Ishida, Jun Adachi, Masayuki Kano, Hisahiro Matsubara, Takeshi Tomonaga

**Affiliations:** 1 Laboratory of Proteome Research, National Institute of Biomedical Innovation, Health and Nutrition, Osaka, Japan; 2 Department of Frontier Surgery, Graduate School of Medicine, Chiba University, Chiba, Japan

## Abstract

At the moment, there is no sensitive clinical test for detecting early-stage colorectal cancer (CRC). Target proteomics has enabled high-throughput verification of hundreds of biomarker candidate proteins. Using this technology, we verified 725 previously reported CRC biomarker candidate proteins that are functionally correlated with CRC in extracellular vesicles (EVs) from patients. Of these, 356 proteins were quantified, and 34 peptides (22 proteins) showed significant differences in the serum EVs between healthy controls and CRC patients of two independent cohorts (n = 77 and 84). These peptides were evaluated as single or multiple markers, and four single peptides in annexin family proteins and eight combinations of peptides showed area under the curve > 0.9 for discriminating between healthy controls and CRC patients. The sensitivities of annexins A3, A4, and A11 peptides for detecting early-stage CRC greatly exceed those of carcinoembryonic antigen. These peptides are promising biomarkers for early detection of CRC.

## Introduction

Colorectal cancer (CRC) is one of the most frequent cancers in the world and a significant cause of human mortality^1^. Development of effective biomarkers for CRC is essential for improving therapeutic outcomes. Especially, there is a compelling need for blood biomarkers because of their usefulness in examinations. In past decades, large-scale omics studies for biomarker discovery have listed numerous candidates and cancer-related factors^2–4^. Despite finding many biomarker candidates, effective blood biomarkers have not yet been established. In recent years, the US Food and Drug Administration has approved of only a very few biomarkers (≤2 per year)^5^. One cause of this stagnation is that there seems to be a serious bottleneck in the pipeline of biomarker development.

This bottleneck may result from a lack of effective methods for verification assays. Currently, in many clinical studies, verification of protein biomarker candidates is performed using antibody-based quantification assays, mainly enzyme-linked immunosorbent assays (ELISA). Because these assay systems are largely dependent on the quality of antibodies, it is difficult to simultaneously evaluate numerous marker candidates. For that reason, in antibody-based systems, the number of biomarker candidates often must be decreased before the verification assay. So, to evaluate many biomarker candidates, it is necessary to construct another assay system that does not use antibodies.

Recent advances in mass spectrometry (MS) have led to a proposed alternative method for candidate protein verification. Selected reaction monitoring (SRM) is a target proteomics method characterized by high mass resolution and accuracy and is generally performed on a triple-quadrupole mass spectrometer^6^. In a previous report, Whiteaker *et al*. performed quantification of a large number of putative breast cancer biomarker candidates using an SRM method in patient plasma^7^. Their strategy demonstrated the usefulness of SRM for biomarker verification and the possibility of its application to other bioresources and diseases. Previously, our group has also verified more than a hundred breast and colorectal cancer biomarker candidates by using the SRM method^8–10^. Thus, targeted proteomics is a powerful tool for biomarker validation.

However, the dynamic range of blood proteins is extremely large^11^; if there is only a small amount of the candidate protein, detection in total blood protein may be difficult even if SRM is used. Therefore, we focused on extracellular vesicles (EVs) in the blood as a potential biomarker. EVs are secreted from almost all cell types and act as mediators of intracellular communication. Recent studies have reported that EV component proteins had pathological roles in several diseases, including cancer^12^. Therefore, EV proteins are considered to be promising biomarker candidates.

In this study, we selected CRC biomarker candidate proteins from a PubMed literature search. The candidate proteins were quantitated in EV fractions of patient sera by using targeted proteomic analysis. We identified several promising biomarker candidates for early diagnosis of CRC.

## Results

### PubMed search of the CRC related biomarker candidate proteins

**Figure 1:**
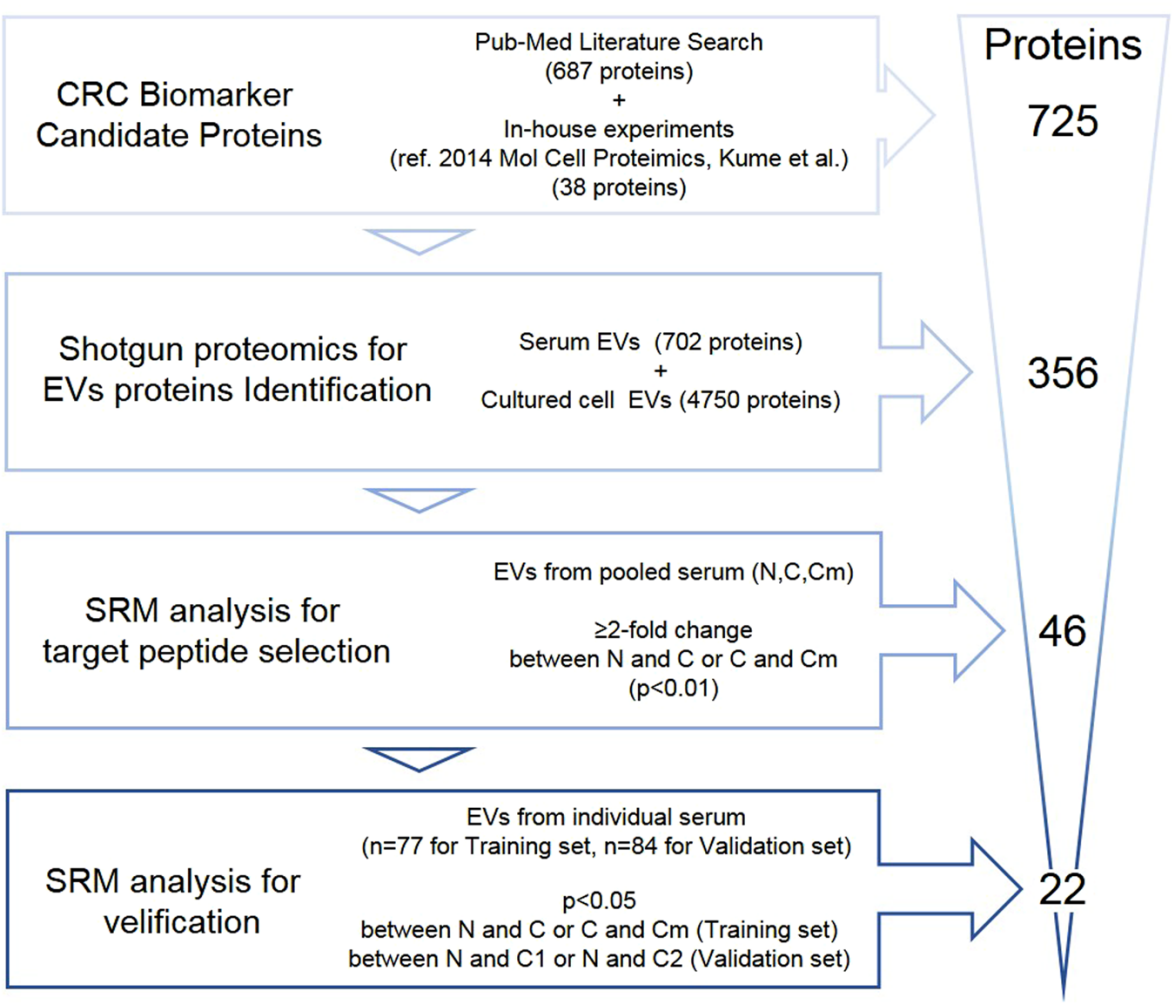
Strategy of CRC biomarker discovery in EVs. Overview of the strategy of biomarker candidate selection. The method of selection at each step and the number of narrowed down candidate proteins is indicated. CRC: Colorectal Cancer, EVs: extracellular vesicles.

The strategy of this study is illustrated in Fig. 1. A list of CRC biomarker candidate proteins was obtained from a PubMed database of the medical and biological literature. A PubMed search from 2003 to 2014 was conducted by using the search query “cancer” AND “colorectal” AND “expression.” A total of 687 proteins had been previously reported in association with CRC. These 687 proteins were listed as the CRC biomarker candidates. We also applied the following inclusion criteria: (1) protein expression had been verified in human CRC tissue or blood by western blot, ELISA, or immunohistochemistry; (2) the targeted protein was upregulated in cancer (upregulated proteins are more suitable as biomarkers than are downregulated proteins); and (3) the molecular function of the targeted protein related to cancer development and progression had been reported or experimentally verified by RNAi or overexpression of the molecule. (4) Simply identified proteins in large-scale analysis (e.g., omics analysis) were excluded. Furthermore, we previously identified 44 CRC biomarker candidate proteins by targeted proteomics of clinical specimens^8^. These 44 proteins were combined with the above biomarker candidates to give a total of 725 proteins, excluding overlapped proteins, that were finally selected as CRC biomarker candidates (Supplementary Table 1).

### Shotgun proteomic analysis for CRC biomarker candidate proteins in EVs

**Figure 2:**
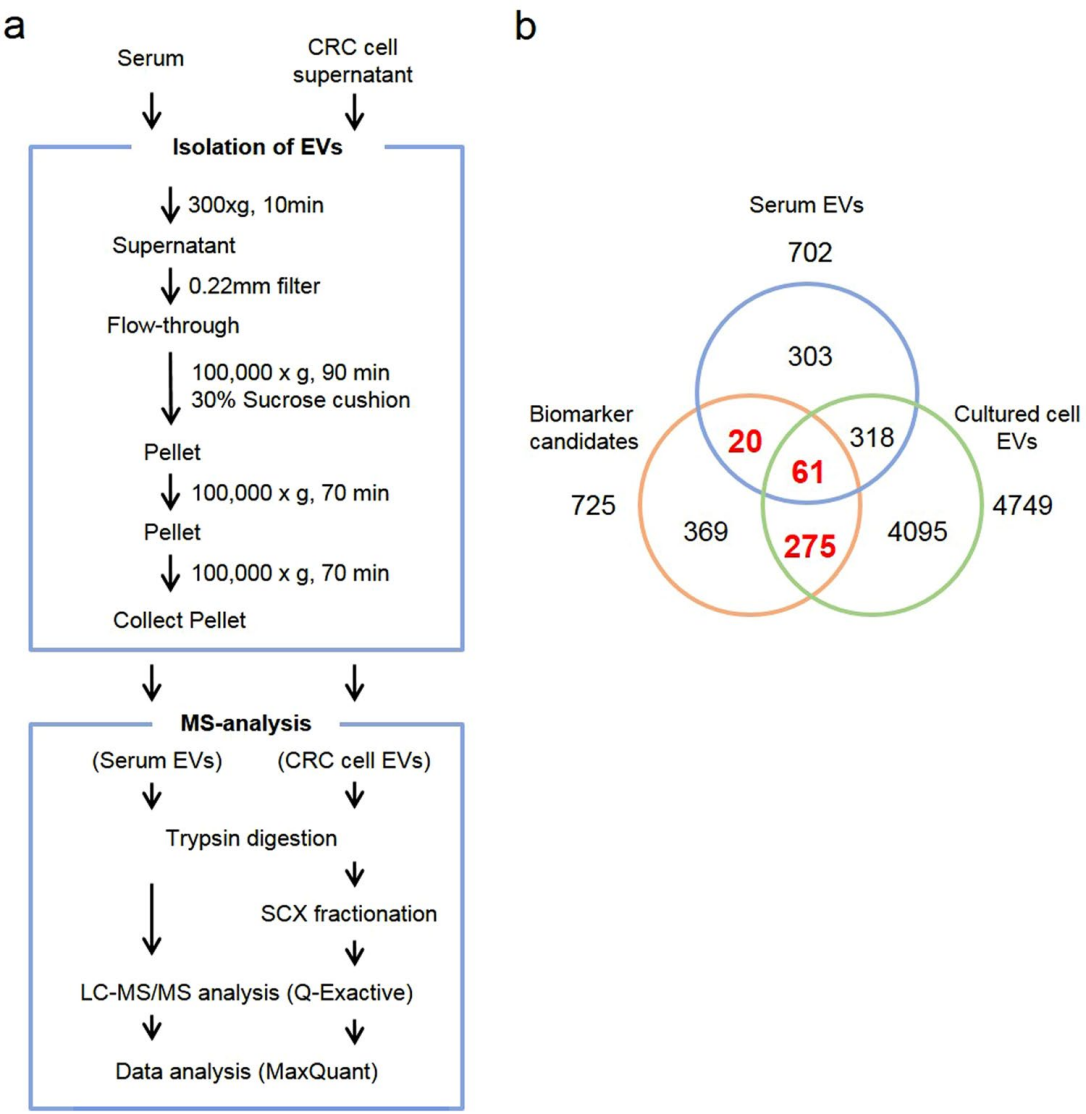
Shotgun proteomics for identification of EV proteins in CRC biomarker candidate proteins. (**a**) Experimental procedure for preparation of EVs and for MS analysis. (**b**) Venn diagram analysis of biomarker candidate proteins and EV proteins identified by shotgun proteomic analysis. Overall, 356 candidate proteins (total of bold red numbers) were identified in EVs from sera or cell culture supernatants. EVs: extracellular vesicles.

Next, we determined how many CRC biomarker candidate proteins in EVs were detected by proteomics. To investigate EV proteins, we performed a shotgun proteomic analysis by using EV fractions prepared from sera and cultured cell supernatants. The shotgun proteomics workflow and specimens are summarized in Fig. 2a and Supplementary Table 2. Preparation of EV fractions from serum and cell supernatant was performed by ultracentrifugation^13^. Serum EVs were collected from eight patients of non-cancer controls and cancers with or without metastasis. Cultured cell supernatants were collected from 4 CRC cells (HCT116; DLD-1, SW480, SW620). Extracted proteins were digested by applying the phase transfer surfactant (PTS) method. Then, digested peptides from four CRC cells EVs were fractionated on a C-18 SCX StageTip column^14,15^. The LC-MS/MS analysis and subsequent mascot database search identified 702 (serum EVs) and 4749 (cell supernatant EVs) proteins, respectively (false discovery rate < 1%, Supplementary Table 3a and b). In the shotgun analysis, 356 proteins were identified as EV proteins among the 725 candidate proteins (Fig. 2b).

### SRM target peptide selection from identified biomarker candidates

To verify candidate protein as biomarkers, we performed SRM analysis of EV fractions from CRC patient sera. First, SRM candidate peptides were selected from all identified peptides in the shotgun proteomics. The following criteria for candidate peptide selection were used: (1) If the identified protein had multiple unique peptides, the target sequences with the highest intensities were selected. (2) Peptides that had missed cleavage or modifications (e.g., oxidized methionine) were excluded. (3) Peptides that were too long (>20 amino acids) were excluded from the target peptide set because construction of a stable isotope-labeled peptide (SI-peptide) was difficult. Among all identified peptides, 3316 peptides (346 proteins) were matched with their criteria (Supplementary Table 4).

Next, to select the SRM target peptides from these candidates, we performed SRM analysis by using EV fractions that were prepared from pooled sera of the non-cancer controls (designated as N, n = 26), pooled sera from cancer without metastasis (designated as C, n = 26), and pooled from cancer with metastasis (designated as Cm, n = 25). For each peptide, three or four transitions (pairs of precursor ion and product ion) with the highest intensities obtained from the shotgun analysis were selected. SRM analyses were performed in triplicate for each pooled fraction, and peptide peak areas were quantified by using Skyline software. We then selected target peptides whose peak areas were quantifiable and increased more than two-fold (p < 0.01) between stages (N vs. C or C vs. Cm). For candidate proteins with ≥ 2 target peptides, we selected the two peptides with the highest intensities as the next step target. Considering these criteria, 71 peptides (46 proteins) were identified as target peptides in this assay (Table 1).

**Table 1:** List of SRM target proteins and peptides.

ID	Protein Name	Peptide 1	Peptide 2
P31947	14-3-3 protein sigma	GEELSCEER	YLAEVATGDDK
P11021	78 kDa glucose-regulated protein	SDIDEIVLVGGSTR	VLEDSDLK
P02763	Alpha-1-acid glycoprotein 1	YVGGQEHFAHLLILR	EQLGEFYEALDCLR
Q15389	Angiopoietin-1	LEIQLLENSLSTYK	ENLQGLVTR
P50995	Annexin A11	GTITDAPGFDPLR	SETDLLDIR
P12429	Annexin A3	GAGTNEDALIEILTTR	SDTSGDYEITLLK
P09525	Annexin A4	GAGTDEGCLIEILASR	DEGNYLDDALVR
Q8N6Q3	CD177 antigen	GGGIFSNLR	QIGIFSAR
P31997	CD67 antigen	EVLLLVHNLPQDPR	LFIPNITTK
P21730	CD88 antigen	SLPSLLR	NVLTEESVVR
P01024	Complement C3	TGLQEVEVK	AAVYHHFISDGVR
P02748	Complement component C9	AIEDYINEFSVR	LSPIYNLVPVK
P27487	Dipeptidyl peptidase 4	IISNEEGYR	IQLSDYTK
P46934	E3 ubiquitin-protein ligase NEDD4	DFVLHPR	FIIDEELFGQTHQHELK
P55060	Exportin-2	TGNIPALVR	SANVNEFPVLK
P49327	Fatty acid synthase	EGGFLLLHTLLR	FPQLDSTSFANSR
P11166	GLUT-1	VTILELFR	TFDEIASGFR
P62993	Growth factor receptor-bound protein 2	YFLWVVK	NQQIFLR
P04792	Heat shock protein beta-1	QDEHGYISR	DGVVEITGK
P14780	Matrix metalloproteinase-9	QSTLVLFPGDLR	FQTFEGDLK
Q9HC84	Mucin-5B	VCGLCGNFDDNAINDFATR	AAGGAVCEQPLGLECR
P31949	Protein S100-A11	DGYNYTLSK	CIESLIAVFQK
P06702	Protein S100-A9	LGHPDTLNQGEFK	DLQNFLK
Q9UQP3	Tenascin-N	AQTEIDGPK	EEQNIIFR
P02786	Transferrin receptor protein 1	LLNENSYVPR	VSASPLLYTLIEK
P08758	Annexin A5	SEIDLFNIR	
Q15717	ELAV-like protein 1	NVALLSQLYHSPAR	
P60228	Eukaryotic translation initiation factor 3 subunit E	LFIFETFCR	
Q96PY5	Formin-like protein 2	VEELEENISHLSEK	
P06396	Gelsolin	EVQGFESATFLGYFK	
P54652	Heat shock-related 70 kDa protein 2	NALESYTYNIK	
P05362	Intercellular adhesion molecule 1	VELAPLPSWQPVGK	
P50579	Methionine aminopeptidase 2	HLLNVINENFGTLAFCR	
O15427	Monocarboxylate transporter 4	AVSVFFK	
Q15758	Neutral amino acid transporter B(0)	GPAGDATVASEK	
P80188	Neutrophil gelatinase-associated lipocalin	ELTSELK	
O00592	Podocalyxin	ATFNPAQDK	
P28066	Proteasome subunit alpha type-5	PFGVALLFGGVDEK	
P05109	Protein S100-A8	ALNSIIDVYHK	
P00352	Retinal dehydrogenase 1	TIPIDGNFFTYTR	
P36952	Serpin B5	ELETVDFK	
P42224	Signal transducer and activator of transcription 1-alpha/beta	FNILGTHTK	
P24557	Thromboxane-A synthase	EAAQDCEVLGQR	
O14773	Tripeptidyl-peptidase 1	LFGGNFAHQASVAR	
P12956	X-ray repair cross-complementing protein 6	DSLIFLVDASK	
Q6UX06	Olfactomedin-4	VQSINYNPFDQK	

### Assessment of the quality of the quantitative data by SRM analysis

#### Transitions consistency assessment of SRM analysis using technical replicates

Reproducibility of data is important for multi-sample measurements. To maximize the quality of the data obtained by SRM analysis, we examined the experimental accuracy of the optimized transition and acquisition parameters by using technical replicates. SI-peptides for 71 target peptides were synthesized as internal standards. For each peptide, 3 or 4 transitions were selected. Collision energy optimization was performed by using an SI-peptide mixture. To distinguish an endogenous peak with a non-specific background, we compared the peaks of endogenous and SI-peptides. Skyline software determined the peak similarity between endogenous and SI-peptides and scored it as the “dotp ratio”^16^. We adopted a dotp > 0.9 as the threshold value for endogenous peak detection. Furthermore, we confirmed the transition consistency of SRM analysis by using three technical replicates of identical serum EV samples. The coefficient of variation (CV) of the peak areas of the transitions was < 30%, which indicated that the selected transitions were reproducible (Supplementary Table 5).

#### Assessment of the reproducibility of the EV protein extraction and protein digestion procedures by using biological replicates

There are various methods for collecting EVs, and none has been confirmed. In this study, EV purification was performed by ultracentrifugation using a sucrose cushion. Compared with other commercially available purification kits, this method gives higher purity of the isolated EVs (data not shown); however, it is uncertain if the procedure is reproducible. Therefore, we verified the reproducibility of our EV purification method together with the protein extraction and digestion procedures. Three replicates of sample preparations from identical serum pools were used. Each sample was analyzed in duplicate, and the quantitative value of the target peptide was calculated from the total peak area ratio of each transition of endogenous peptides to that of the SI-peptides. The CVs of the quantification values for EV marker peptides (CD9 and CD81) were < 16.5% (Supplementary Table 6). Thus, our EV collection experiment was accurately reproduced.

### Verification of biomarker candidate proteins by SRM

Next, we performed an SRM verification assay of selected biomarker candidate peptides in the EV fractions prepared from individual patient serum. Individual EV fractions were prepared from three groups of patient sera (N, n = 26; C, n = 26; Cm, n = 25). Then, SRM analyses were performed in technical duplicates for each sample. A two-tailed non-paired t-test showed that a total of 37 peptides (22 proteins) were significantly increased in C relative to N and in Cm relative to N and C (p < 0.05) (Table 2, Supplementary Figure 1).

**Table 2:** List of verified peptides with high or moderate (bold; AUC > 0.7) accuracy. (N.S.: not significant).

	Protein Name	Accession	Peptide	p-value		AUC	
				N vs C	C vs Cm	N and C	C and Cm
1	Annexin A11	P50995	GTITDAPGFDPLR	P < 0.01	N.S.	**0.99**	0.53
			SETDLLDIR	P < 0.01	N.S.	**0.97**	0.54
2	Annexin A3	P12429	GAGTNEDALIEILTTR	P < 0.01	P < 0.01	**0.84**	**0.75**
			SDTSGDYEITLLK	P < 0.01	N.S.	**0.95**	0.58
3	Annexin A4	P09525	GAGTDEGCLIEILASR	P < 0.01	P < 0.05	**0.82**	**0.70**
			DEGNYLDDALVR	P < 0.01	N.S.	**0.96**	0.60
4	Tenascin-N	Q9UQP3	EEQNIIFR	P < 0.01	N.S.	**0.85**	0.57
			AQTEIDGPK	P < 0.01	N.S.	**0.85**	0.55
5	Transferrin receptor protein 1	P02786	LLNENSYVPR	P < 0.01	N.S.	**0.85**	0.50
			VSASPLLYTLIEK	P < 0.01	N.S.	**0.74**	0.61
6	GLUT-1	P11166	VTILELFR	P < 0.05	P < 0.05	0.67	**0.71**
			TFDEIASGFR	P < 0.01	N.S.	**0.88**	0.53
7	Complement component C9	P02748	LSPIYNLVPVK	P < 0.01	P < 0.05	**0.81**	0.64
			AIEDYINEFSVR	P < 0.05	P < 0.05	**0.74**	0.63
8	CD88 antigen	P21730	SLPSLLR	P < 0.05	N.S.	**0.71**	0.59
			NVLTEESVVR	P < 0.01	P < 0.01	**0.78**	**0.71**
9	78 kDa glucose-regulated protein	P11021	VLEDSDLK	P < 0.01	P < 0.05	**0.74**	0.63
			SDIDEIVLVGGSTR	P < 0.05	P < 0.01	**0.71**	**0.72**
10	Alpha-1-acid glycoprotein 1	P02763	YVGGQEHFAHLLILR	N.S.	P < 0.05	0.51	**0.70**
			EQLGEFYEALDCLR	N.S.	P < 0.05	0.60	0.62
11	Matrix metalloproteinase-9	P14780	QSTLVLFPGDLR	N.S.	P < 0.01	0.59	**0.72**
			FQTFEGDLK	P < 0.01	N.S.	**0.87**	0.52
12	Angiopoietin-1	Q15389	LEIQLLENSLSTYK	N.S.	P < 0.05	0.55	0.68
			ENLQGLVTR	P < 0.01	N.S.	**0.77**	0.53
13	CD67 antigen	P31997	EVLLLVHNLPQDPR	N.S.	P < 0.01	0.56	**0.83**
			LFIPNITTK	P < 0.01	P < 0.01	**0.70**	**0.70**
14	Mucin-5B	Q9HC84	AAGGAVCEQPLGLECR	N.S.	P < 0.05	0.61	0.67
			VCGLCGNFDDNAINDFATR	N.S.	P < 0.01	0.64	**0.85**
15	Adapter protein GRB2	P62993	YFLWVVK	N.S.	P < 0.05	0.51	0.68
			NQQIFLR	P < 0.01	N.S.	**0.74**	0.56
16	Annexin A5	P08758	SEIDLFNIR	P < 0.01	N.S.	**0.84**	0.61
17	Olfactomedin-4	Q6UX06	VQSINYNPFDQK	P < 0.01	N.S.	**0.78**	0.50
18	Neutral amino acid transporter B(0)	Q15758	GPAGDATVASEK	P < 0.05	N.S.	**0.77**	0.55
19	Tripeptidyl-peptidase 1	O14773	LFGGNFAHQASVAR	P < 0.01	P < 0.05	**0.72**	0.65
20	Heat shock-related 70 kDa protein 2	P54652	NALESYTYNIK	P < 0.05	N.S.	0.53	0.64
21	Proteasome subunit alpha type-5	P28066	PFGVALLFGGVDEK	N.S.	P < 0.05	0.63	**0.71**
22	Neutrophil gelatinase-associated lipocalin	P80188	ELTSELK	P < 0.01	N.S.	**0.78**	0.53

### Statistical analysis and evaluation of target peptides as CRC biomarkers

**Figure 3:**
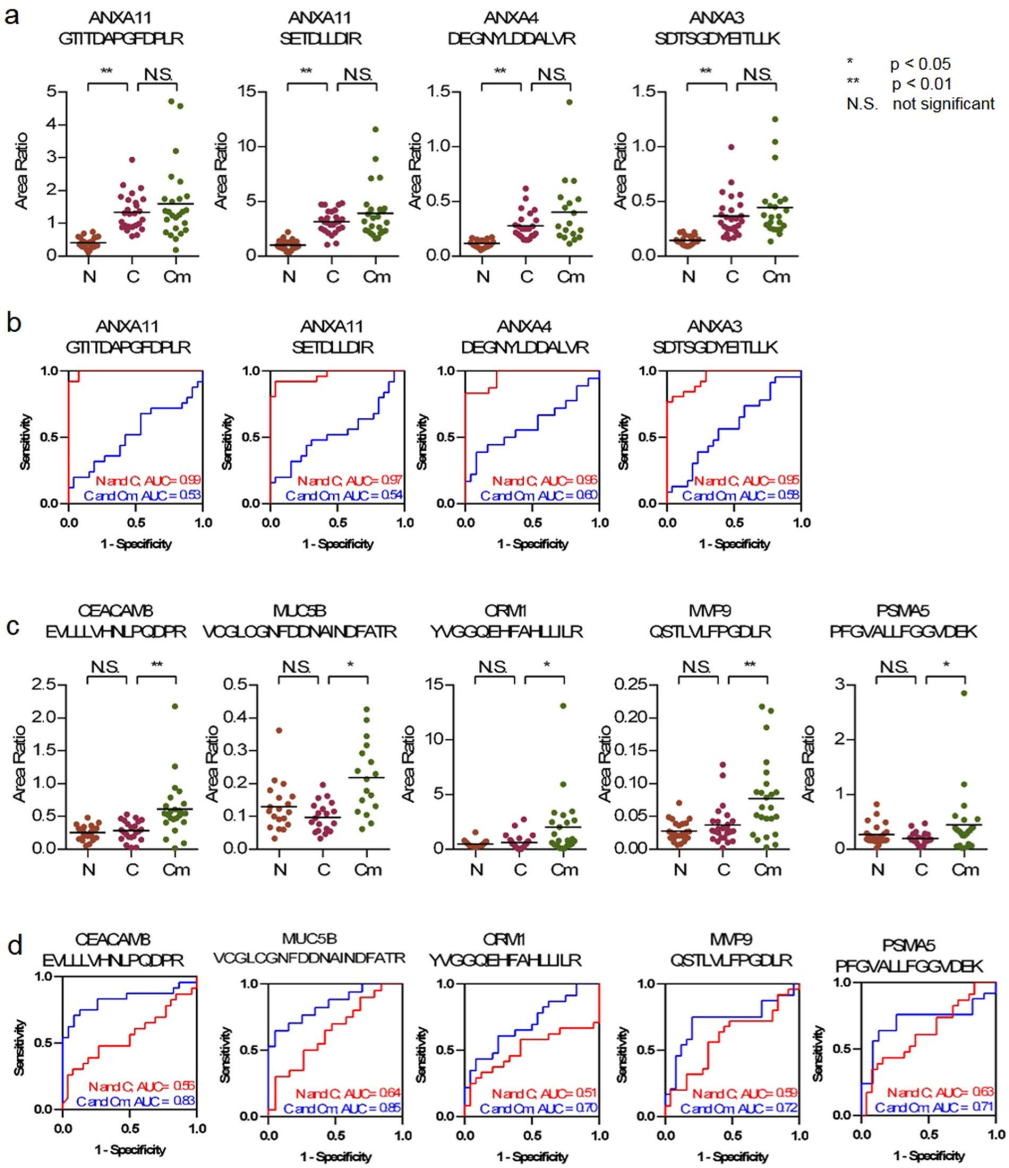
Statistical analysis of target peptides. (**a**,**c**) Relative quantitation of peptides between three cohorts by SRM analysis (N: non-cancer control, C: cancer without metastasis, Cm:Cancer with metastasis). Graph of dot plot shows the peak area ratio of the endogenous peptide to that of the SI-peptide. (*p < 0.05, **p < 0.01, N.S: not significant) (**b**,**d**) ROC curve analysis for discriminating between N and C (red line) or between C and Cm (blue line). The area under the curve (AUC) for the discrimination is shown on each graph. SRM: Selected reaction monitoring, ROC: Receiver Operating Characteristic.

To determine whether the candidate peptides were as efficient as CRC biomarkers, we performed receiver operating characteristic (ROC) analysis of the verified 37 peptides. Discrimination between N and C and between C and Cm for each candidate peptide was evaluated. Four peptides (3 proteins) were highly sensitive [area under the curve (AUC) > 0.9, Fig. 3a,b], and 22 peptides were moderately sensitive (AUCs from 0.7–0.9, Supplementary Figure 2) for discrimination between N and C, whereas 11 peptides were moderately sensitive for discrimination between C and Cm (Fig. 3c,d, Supplementary Figure 2).

**Figure 4:**
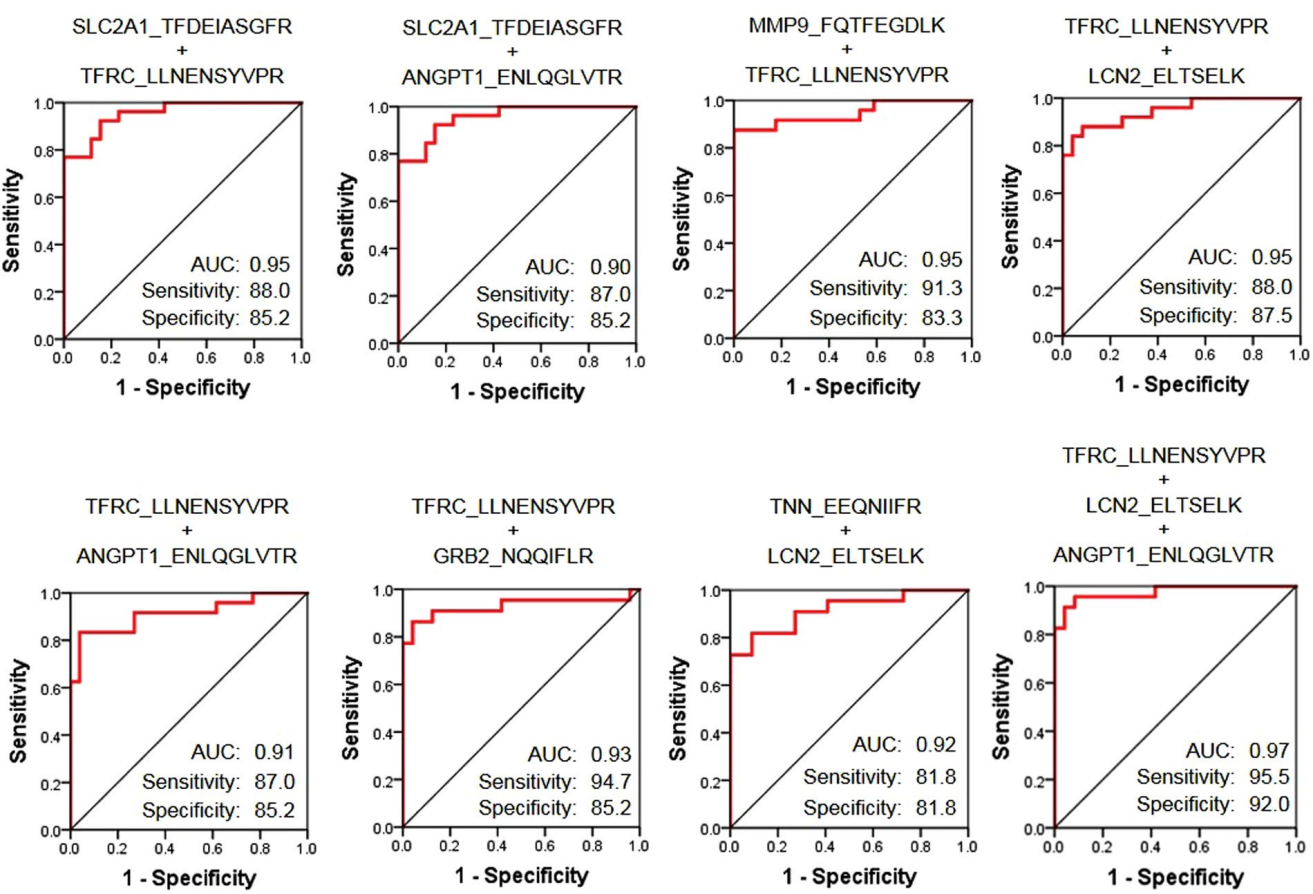
ROC curve analysis for combination of target peptides. The diagnostic sensitivity between N and C of the peptide combination was evaluated. The area under the curve (AUC), sensitivity, and specificity are shown on each graph. High-accuracy combinations (AUC > 0.9) are shown; other combinations are shown in Supplemental Figure 3.

Next, we investigated whether higher sensitivity could be obtained by a combination of candidate peptides. A logistic regression-based combination of candidate markers was established by using SPSS software. For logistic analysis, highly correlated combinations must be avoided, so before the analysis, we investigated the correlation of candidate peptides. Some peptide sets were highly correlated (R > 0.7) (Supplementary Table 7). Then, logistic analysis with ≥ 2 peptides, excluding correlated peptides, was performed, and ROC curves were generated for each set. The AUCs of 14 combinations of candidate peptides were significantly increased (Supplemental Figure 3), especially the AUCs of eight combinations were > 0.9 (Fig. 4). The highest AUC (0.97) was obtained for a combination of three peptides (transferrin receptor protein 1, neutrophil gelatinase-associated lipocalin, and angiopoietin-1). These results suggested that combinations of multiple markers could improve the accuracy of early diagnosis of CRC.

### Comparison of the sensitivities of newly identified target peptides with that of a widely used CRC tumor marker, carcinoembryonic antigen (CEA), for early detection of CRC

**Figure 5:**
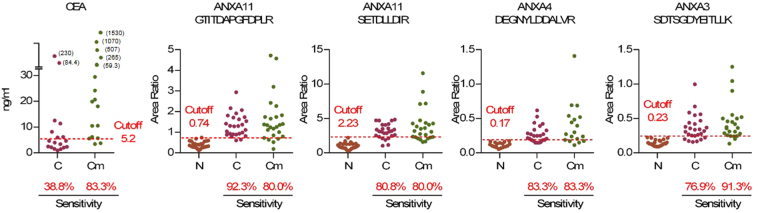
Comparison of the sensitivities of target peptides with that of CEA. Sensitivity is calculated as the percentage of sample when the cutoff value is set at the maximum peak area of N (red dashed line; specificity = 100%). In this figure, the top 3 peptides in sensitivity for discriminating between N and C are shown. Other peptides that had greater sensitivities than that of CEA are shown in Supplemental Figure 4. CEA: carcinoembryonic antigen.

CEA is one of the most widely used blood CRC markers today. However, the sensitivity of CEA is insufficient for detection of early-stage CRC patients. It has been reported that only 30% of CRC stage two patients can be diagnosed^17^. Here we compared the sensitivity of the verified biomarker candidates described above with that of CEA. The cutoff points of each candidate peptide for the SRM results were set to 100% specificity because the specificity of CEA is almost 100%^18^. The sensitivity of CEA for specimen group C was 38.8%; on the other hand, the sensitivities of 17 peptides (12 proteins; annexin A3, annexin A4, annexin A5, annexin A11, tenascin-N, transferrin receptor protein 1, GLUT-1, matrix metalloproteinase-9, olfactomedin-4, CD88 antigen, tripeptidyl-peptidase 1, and neutrophil gelatinase-associated lipocalin) were largely surpassed CEA (Supplementary Figure 4). Among them, the sensitivities of four peptides of annexins A3, A4, and A11 were > 75% (Fig. 5).

### Validation of biomarker candidate proteins in another cohort

**Figure 6:**
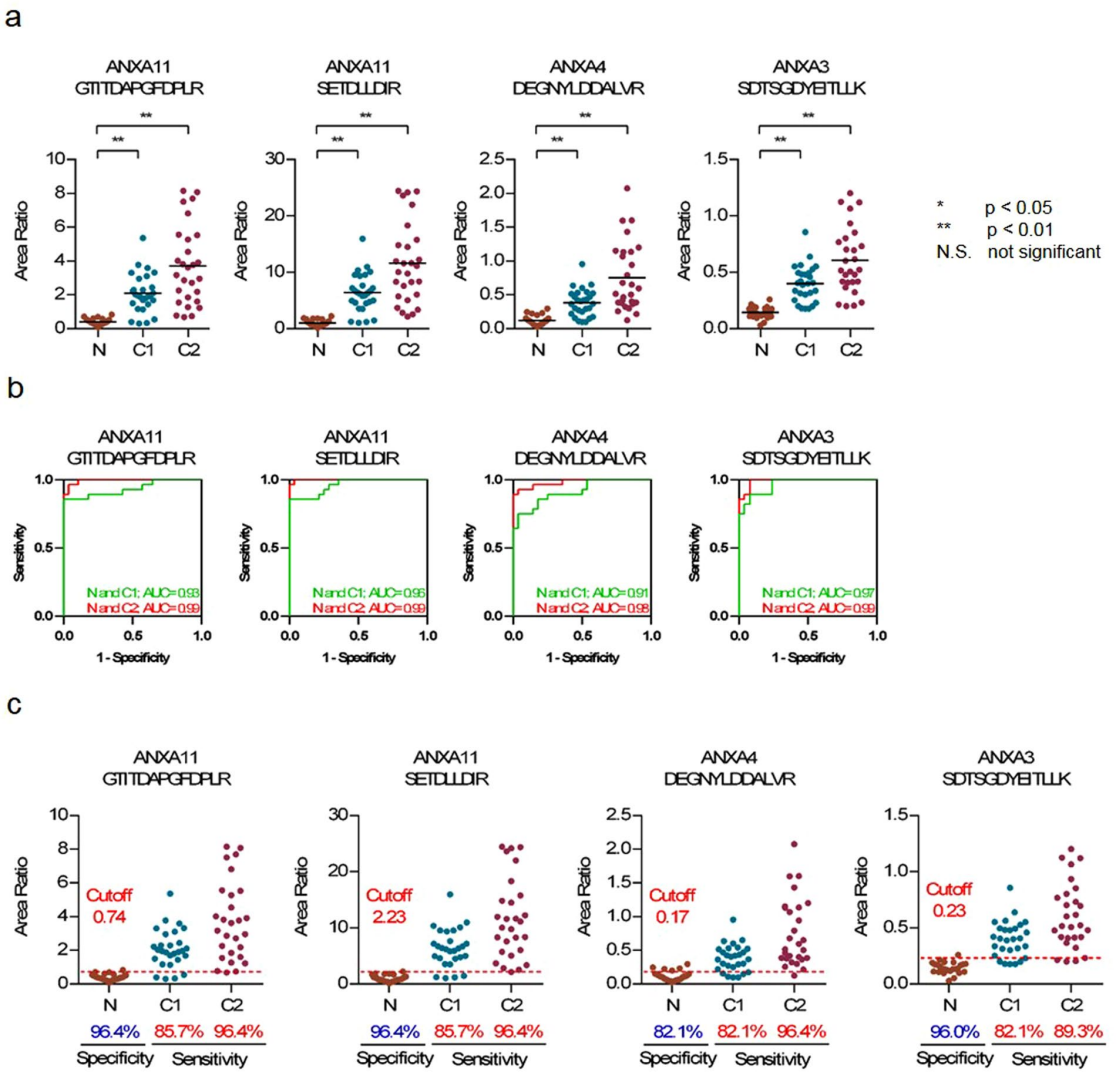
Validation of candidate peptides in another cohort. (**a**) Relative quantitation of peptides among three cohorts by SRM analysis (N: non-cancer control, C1: stage1 cancer, C2: stage2 cancer). Graph of dot plot shows the peak area ratio of the endogenous peptide to that of the SI-peptide (*p < 0.05, **p < 0.01, N.S: not significant) (**b**) ROC curve analysis for discriminating between N and C1 (green line) or between N and C2 (red line). The area under the curve (AUC) for the discrimination is shown on each graph. (**c**) Sensitivities and specificities of target peptides in another cohort. Sensitivity and specificity are calculated as the percentage of sample using the cutoff point obtained by the training study.

Next, we verified the candidate peptides using another cohort of healthy controls and stage 1 (C1) and stage 2 CRC (C2). Individual EV fractions were prepared from three groups of patient sera (N, n = 28; C1, n = 28; C2, n = 28). Then, SRM analyses were performed in technical duplicates for each sample. In this SRM assay, two candidate peptides (SLPSLLR and FQTFEGDLK) could not be detected in more than half of the specimens. Therefore, it was verified that 34 of the 37 candidate peptides (22 proteins) were significantly increased (p < 0.05) in another cohort of stage 2 cancer specimens by a two-tailed non-paired t-test. Furthermore, it was confirmed that 33 peptides (22 proteins) were significantly increased (p < 0.05) in the specimens of the stage 1 cancer patient group (Table 3, Fig. 6a, and Supplementary Figure 5). ROC analysis indicated that the 33 peptides were moderately (AUC > 0.7) or highly (AUC > 0.9) sensitive between N and C1 or C2 (Fig. 6b, Supplementary Figure 6).

**Table 3:** Results of verification of candidate peptides in another cohort. (N.S.: not significant, N.D.: not detected, bold; AUC > 0.7.).

	Protein Name	Accession	Peptide	p-value		AUC	
				N vs C1	N vs C2	N and C1	N and C2
1	Annexin A11	P50995	GTITDAPGFDPLR	P < 0.01	P < 0.01	**0.93**	**0.99**
			SETDLLDIR	P < 0.01	P < 0.01	**0.96**	**0.99**
2	Annexin A3	P12429	GAGTNEDALIEILTTR	P < 0.01	P < 0.01	**0.99**	**0.99**
			SDTSGDYEITLLK	P < 0.01	P < 0.01	**0.97**	**0.99**
3	Annexin A4	P09525	GAGTDEGCLIEILASR	P < 0.01	P < 0.01	**0.94**	**0.97**
			DEGNYLDDALVR	P < 0.01	P < 0.01	**0.91**	**0.98**
4	Tenascin-N	Q9UQP3	EEQNIIFR	P < 0.01	P < 0.01	**0.78**	**0.79**
			AQTEIDGPK	P < 0.01	P < 0.01	**0.77**	**0.78**
5	Transferrin receptor protein 1	P02786	LLNENSYVPR	P < 0.01	P < 0.01	**0.85**	**0.88**
			VSASPLLYTLIEK	P < 0.01	P < 0.01	**0.91**	**0.92**
6	GLUT-1	P11166	VTILELFR	P < 0.01	P < 0.01	**0.96**	**0.96**
			TFDEIASGFR	P < 0.01	P < 0.01	**0.93**	**0.98**
7	Complement component C9	P02748	LSPIYNLVPVK	P < 0.01	P < 0.01	**0.91**	**0.98**
			AIEDYINEFSVR	P < 0.01	P < 0.01	**0.81**	**0.95**
8	CD88 antigen	P21730	SLPSLLR	N.D.	N.D.	N.D.	N.D.
			NVLTEESVVR	P < 0.01	P < 0.01	**0.94**	**0.98**
9	78 kDa glucose-regulated protein	P11021	VLEDSDLK	P < 0.01	P < 0.01	**0.86**	**0.92**
			SDIDEIVLVGGSTR	P < 0.05	P < 0.01	**0.78**	**0.85**
10	Alpha-1-acid glycoprotein 1	P02763	YVGGQEHFAHLLILR	P < 0.01	P < 0.01	**0.88**	**0.89**
			EQLGEFYEALDCLR	P < 0.01	P < 0.01	**0.82**	**0.85**
11	Matrix metalloproteinase-9	P14780	QSTLVLFPGDLR	P < 0.01	P < 0.01	**0.97**	**0.99**
			FQTFEGDLK	N.D.	N.D.	N.D.	N.D.
12	Angiopoietin-1	Q15389	LEIQLLENSLSTYK	N.S.	N.S.	0.67	0.55
			ENLQGLVTR	P < 0.01	P < 0.01	**0.74**	**0.75**
13	CD67 antigen	P31997	EVLLLVHNLPQDPR	P < 0.01	P < 0.01	**0.98**	**0.99**
			LFIPNITTK	P < 0.01	P < 0.01	**0.98**	**0.99**
14	Mucin-5B	Q9HC84	AAGGAVCEQPLGLECR	N.S.	P < 0.01	0.64	**0.79**
			VCGLCGNFDDNAINDFATR	P < 0.05	P < 0.01	**0.74**	**0.88**
15	Adapter protein GRB2	P62993	YFLWVVK	P < 0.01	P < 0.01	**0.83**	**0.94**
			NQQIFLR	P < 0.01	P < 0.01	**0.85**	**0.96**
16	Annexin A5	P08758	SEIDLFNIR	P < 0.01	P < 0.01	**0.98**	**0.99**
17	Olfactomedin-4	Q6UX06	VQSINYNPFDQK	P < 0.01	P < 0.01	**0.95**	**0.98**
18	Neutral amino acid transporter B(0)	Q15758	GPAGDATVASEK	P < 0.01	P < 0.01	**0.87**	**0.84**
19	Tripeptidyl-peptidase 1	O14773	LFGGNFAHQASVAR	P < 0.01	P < 0.01	**0.84**	**0.88**
20	Heat shock-related 70 kDa protein 2	P54652	NALESYTYNIK	P < 0.01	P < 0.01	**0.87**	**0.89**
21	Proteasome subunit alpha type-5	P28066	PFGVALLFGGVDEK	P < 0.01	P < 0.05	**0.79**	0.66
22	Neutrophil gelatinase-associated lipocalin	P80188	ELTSELK	P < 0.01	P < 0.01	**0.95**	**0.95**

To verify whether these candidate peptides are better biomarkers than CEA, we validated the diagnostic value of the cutoff point obtained by the training study in another cohort. Area ratio values of the two cohort studies were normalized by the average area ratio of healthy controls. As a result, the sensitivities of annexins A3, A4, and A11 were 82.1–85.7% in stage 1 patients and 89.3–96.4% in stage 2 patients, whereas the specificities were 82.1–96.4% (Fig. 6c). Moreover, the sensitivities of 22 and 28 peptides exceed those of CEA in stage 1 and stage 2 patients, respectively, and the specificities of all the peptides exceed 80% (Supplementary Figure 7). Therefore, we could successfully verify the results obtained in the training cohort.

## Discussion

Development of early diagnostic markers is indispensable for improving the clinical outcomes of CRC. Currently, widely used tumor markers (e.g., CEA, CA19-9) have low sensitivity for detection of early-stage CRC^18^. To address this problem, we focused on CRC biomarker candidate proteins that have been reported to be functionally correlated to the pathogenesis of CRC. Next, we verified the biomarker candidates in serum EVs by using highly sensitive targeted proteomic technology. Overall, 725 proteins were listed as biomarker candidates, and 37 peptides of 22 proteins were verified as CRC biomarker candidates in EV fractions (Table 2 and Supplementary Figure 1). Surprisingly, four peptides in annexin A3, annexin A4, annexin A11, or combinations of two or three of the 37 peptides were able to discriminate healthy controls from CRC patients with high accuracy (AUC > 0.9) (Figs 3 and 4). Furthermore, statistical analysis of these candidates indicated that the sensitivity of 17 peptides for early diagnosis of CRC exceeded that of the conventional CRC biomarker CEA; especially, the sensitivities of three peptides of annexin A4 and A11 far exceeded the sensitivity of CEA, by > 80% when the specificity was set to 100%, (Fig. 5, Supplementary Figure 3). Similar results were obtained by a validation study using another cohort (Fig. 6, Supplementary Figures 5–7).

EVs are increasingly being considered to be promising biomarker resources because many functional molecules are stably enclosed in the small vesicles. These EVs are thought to be responsible for intercellular communication and important for pathogenesis of various disease^12,19^. Several groups have reported that cancer biomarker candidate proteins are present in EVs. Melo *et al*. reported that glypican-1 in cancer-derived EVs is an early diagnostic marker for pancreatic cancer^20^. Biomarker candidates in EVs have also been reported in other cancers^21–23^. Proteomic analysis for biomarker discovery in EVs has been actively performed. Sequeiros *et al*. recently identified prostate cancer biomarkers in urinary EVs and verified biomarker candidates by using target proteomic technology^24^. Based on these backgrounds, we attempted to search for novel CRC biomarkers in EVs from the sera of patients.

Comprehensive exploration of cancer biomarkers in EVs prepared from patient body fluids is potentially problematic because it is unknown whether the EVs are derived from a tumor. Therefore, we developed a strategy to search for biomarker candidate proteins in EVs that are likely derived from CRC cells. We first conducted a literature search for biomarker candidate proteins that had been functionally validated by knockdown or overexpression experiments using CRC cell lines. These candidate proteins had also been confirmed to be overexpressed in CRC tissues, so the amount of marker protein in EVs could reflect the pathology of cancer.

In this study, we performed verification analysis by using a target proteomics approach. Although most previous studies have used antibodies for verification of candidates, it is difficult to verify hundreds of biomarker candidates because the availability of antibodies is quite limited, and the quality of the antibodies is not always sufficient. In contrast, SRM analysis has made it possible to verify almost all candidate proteins with high accuracy and high throughput (≤100 peptides simultaneously). Here, we were able to validate 46 candidate proteins within a single run of SRM analysis and validated 22 proteins as biomarker candidates. Furthermore, because SRM analysis enables quantitation of multiple peptides simultaneously, it can create a multi-biomarker panel. The mechanism of cancer development is complex and diverse^25^, so it is considered that combining multiple markers might be effective for enhancing diagnostic accuracy. In fact, we did improve diagnostic accuracy by combining two or three candidate markers (Fig. 4). In the biomarker exploration research, it is important to verify whether the novel marker candidates are also effective in another cohort. In this study, verification experiments using separate cohorts verified that 33 peptides are very useful for early diagnosis of stage 1 and stage 2 CRC (Supplementary Figures 5–7).

We found that annexin family proteins (annexin A3, A4, A5, and A11) exhibited high sensitivity as CRC biomarkers. Annexins are multifunctional phospholipid-binding proteins that are involved in various biological processes (e.g., apoptosis, cell division, ion transport)^26^. Furthermore, the expression levels of annexin family proteins reportedly are well correlated with prognosis in CRC tissues^27^. In addition, EVs contain abundant annexin family proteins^28^. Thus, the importance of annexin family proteins as CRC biomarker candidates demonstrated in this study supports the previously reported significance of these proteins.

In conclusion, we identified a number of promising CRC biomarker candidate proteins for detection of early-stage CRC. In the near future, development of a high-throughput detection system for verification of candidates as CRC biomarkers by using a larger number of specimens is needed. Targeted proteomic technologies, such as SRM, could replace the most widely used clinical tests for CRC, such as the fecal occult blood test or CEA, and would be powerful tools for early diagnosis of CRC.

## Methods

### Colorectal cancer sera and cultured cells

Colorectal cancer and control sera were obtained from 107 patients and 54 healthy volunteers at the Chiba University School of Medicine and stored at −80  °C until analyses. Informed consent was obtained from all donors, and the protocol was approved by the ethics committees of the National Institute of Biomedical Innovation Health and Nutrition and the Chiba University School of Medicine. All methods were performed in accordance with relevant guidelines and regulations. The clinical information of CRC patients and healthy controls as well as pathological information, including tumor size, tumor location, and tumor depth, are shown in Supplementary Table 2. Four human colorectal carcinoma cell lines HCT116 (ATCC; CCL-247), DLD-1 (ATCC; CCL-221), SW480 (ATCC; CCL-228), and SW620 (ATCC; CCL-227) were grown in RPMI-1640 (Gibco Laboratories) medium with 10% fetal bovine serum (FBS) and antibiotics. Cells were maintained at 37 °C in an incubator supplemented with 5% CO_2_ until they grew to sub-confluence. Then, these cultured cells were washed with FBS-free medium and fresh FBS-free medium was added. After 48 hours, the conditioned medium was collected and subjected to EV isolation.

### Isolation of extracellular vesicles

EVs were isolated from cell-conditioned media or sera by using differential ultracentrifugation and a sucrose cushion^13,29^. In brief, serum or cultured cell supernatants were centrifuged at 300 × g for 10 min to remove larger debris. Then, the supernatants were passed through a 0.22-μm spin filter (Agilent Technologies, Santa Clara, CA) and were centrifuged on a 30% sucrose/D_2_O cushion at 100,000 × g for 90 min. The collected cushion was subsequently ultra-centrifuged at 100,000 × g for 70 min twice.

### Protein extraction from EVs and digestion

Protein extraction and proteolytic digestion were performed by using a PTS protocol^14^. Extracted exosomal proteins were lysed by using a MPEX PTS reagent kit (GL Science, Tokyo, Japan), reduced with 5 mM DTT, and alkylated with 20 mM iodoacetamide. Then, the sample was digested at 37 °C overnight with 1% (w/w) of trypsin (proteomics grade; Roche Mannheim, Germany). After digestion, an equal volume of ethyl acetate was added to the digested samples, the mixtures were acidified with 1% trifluoroacetic acid, and vortexed to transfer detergents to the organic phase. After centrifugation, the aqueous phase containing the peptides was collected and desalted by using Stage Tips^30^.

### Liquid chromatography (LC)–mass spectrometry (MS)/MS and proteomic data analysis

Digested peptides were separated into seven fractions by using a C18-SCX StageTip chromatography column^15^ and then analyzed by using a Q-Exactive mass spectrometer (Thermo Scientific, Bremen, Germany) with an UltiMate 3000 Nano-flow high-performance LC (HPLC) system (Dionex, Sunnyvale, CA) and an HTC-PAL autosampler (CTC Analytics, Zwingen, Switzerland). The analytical column was packed with reverse-phase material ReproSil-Pur C18-AQ, 1.9-μm resin (Dr. Maisch, Ammerbuch-Entringen, Germany) into a self-pulled needle (300 mm length × 75 μm inner diameter). The mobile phases consisted of buffer A (0.1% formic acid and 2% acetonitrile) and B (0.1% formic acid and 90% acetonitrile). Digested peptides were dissolved in buffer A and loaded onto a trap column (0.075 × 20 mm, Acclaim PepMap RSLC Nano-Trap Column; Thermo Scientific). The nano-LC gradient was delivered at 280 nL/min and consisted of a linear gradient of buffer B developed from 5–35% B over 120 min. Full MS scans were performed by using an orbitrap mass analyzer (scan range, 350–1800 m/z, with a resolution of 70 000 after accumulation of ions to a 3 × 10^6^ target value). The ten most intense precursor ions were selected and fragmented in the octopole collision cell by higher-energy collisional dissociation with a maximum injection time of 120 ms and a resolution of 35 000. The MS/MS ion-selection threshold was set to 5 × 10^4^ counts. A 3.0-Da isolation width was chosen. Raw data files were processed by MaxQuant software (version 1.5.1.2). Peak lists were searched against the UniProt Human protein database by using the Andromeda search engine^31^. The precursor mass tolerance was set to 7 ppm, and the fragment ion mass tolerance was set to 0.01 Da. Peptides and proteins were accepted with a false discovery rate of < 1%, which was estimated on the basis of the number of accepted hits from the reverse database.

### LC-SRM analysis

The procedures of SRM analysis were performed as previously described^8–10^. The digested peptides were dissolved in a 2% acetonitrile solution containing 0.1% trifluoroacetic acid and then analyzed by using a TSQ-Vantage triple quadruple mass spectrometer (Thermo Fisher Scientific, Bremen, Germany) with a nano-LC interface (AMR, Tokyo, Japan), Paradigm MS2 (Michrom BioResources, Auburn, CA), and an HTC-PAL autosampler (CTC Analytics, Zwingen, Switzerland). The analytical column was packed with a reversed-phase material (ReproSil-Pur C18-AQ, 1.9-μm resin; Dr. Maisch, Ammerbuch-Entringen, Germany) into a self-pulled needle (100-mm length × 75-μm inner diameter). The mobile phases consisted of buffer A (0.1% formic acid and 2% acetonitrile) and B (0.1% formic acid and 90% acetonitrile). Digested peptides were dissolved in buffer A and loaded onto a trap column (0.075 × 20 mm; Acclaim PepMap RSLC Nano-Trap Column; Thermo Scientific). The nano-LC gradient was delivered at 200 nL/min and consisted of a linear gradient of buffer B developed from 5–35% B in 60 min. The parameters of the instrument were set as follows: 0.002 m/z scan width, 0.7 fwhm Q1 resolution, 1 s cycle time, and 1.8- mTorr gas pressure. The collision energy was optimized for every SRM transition, and data acquisition was performed in scheduled SRM mode (time window, 5 min).

### Selection of SRM target peptides and transitions consistency assessment of SRM analysis by using technical replicates

Target peptides of biomarker candidate proteins were selected from peptides identified in the shotgun proteomic analysis. SRM transition lists of each peptide were created from the spectral library of shotgun proteomic data by using Skyline software. Among the identified peptides, the top 2 highest intensity peptides were selected, and the eight most intense fragment ions were selected from the library. Selected peptides and transitions were tested by SRM analysis using pooled EV fractions prepared from sera. The top 3 or 4 intensity transitions with a signal-to-noise ratio (S/N) > 10 were selected from the test results. If there were ≤ 3 transitions with S/N > 10, the peptide was excluded as a candidate. Finally, verification of the selected transition was determined by assessing the similarity of the peak area ratio in each transition between endogenous and stable SI-peptides. This similarity is represented by “dotp” in the Skyline software, and we set a dotp > 0.9 as the threshold for endogenous peptide detection. SI-peptides were synthesized as isotopically labeled C-terminus Arg 13C6 and 15N4 or Lys 13C6 and 15N2 heavy peptides (SpikeTide L; JPT Peptide Technologies, Berlin, Germany) (crude purity). To further confirm the transition consistency, the same EV protein prepared from pooled sera was measured three times, and the CVs of the peak areas of each transition among the technical replicates were calculated by using Skyline software.

### Assessment of reproducibility of EV protein extraction and protein digestion procedure by using biological replicates

The reproducibility of EV protein extraction and protein digestion processes for SRM quantification was evaluated by analyzing three biological replicates. EV protein was prepared from the same pooled serum and digested by applying the PTS method. These technical procedures were performed three times independently. SI-peptide mixtures were then spiked into each digested peptide after which SRM analysis was performed. Targeted peptide quantification was performed by using Skyline software. Quantitative values were calculated from the sum of peak areas of the top 3 or 4 intensity transitions for each peptide, and the CV of three biological replicates was calculated.

### Quantitation of target peptides by SRM using SI-peptide as the internal standard

Quantitation of target peptides by SRM was performed by using SI-peptide as the internal standard. An SI-peptide mixture was spiked into EV fractions prepared from each individual sera. The amount of spiked SI-peptides was adjusted to be close to that of the endogenous peptide by performing a preliminary analysis. EV proteins, which were equivalent to 50 μL of serum, were analyzed in duplicate under the same condition as described in the previous section. The quantitative value of each transition was calculated as the ratio of the peak area with that of the corresponding transition of the SI-peptide, and the quantitative value of each peptide was calculated from the total area ratio of the top 3 or 4 intensity transitions. For samples with S/N < 10 for each peptide, the quantitative values of the peptides were estimated as “no value” and excluded from the statistical analysis.

### Statistical analysis of peak area ratio by SRM analysis

Statistical analysis of SRM data was performed by using SPSS software (Ver. 23) (SPSS Inc., Chicago, IL). The evaluation of target peptides as CRC biomarkers was performed by performing ROC curve analysis, which provided the AUC^32^. All peptides were tested for differences between N and C or between C and Cm, and *P*-values ≤ 0.05 were considered as indicating statistical significance. To evaluate each peptide as part of a multi-marker panel, a logistic regression model was constructed to assess possible combinations of the peptides. To exclude highly correlated peptides from the logistic model, the correlation of the SRM area ratio was examined for all 37 candidate peptides. For the peptides that were less correlated, possible combinations were constructed into the logistic regression model, and the AUCs of the peptide combinations were evaluated by ROC curve analysis.

## Electronic supplementary material

Supplementary Figure 1–7

Supplementary Table 1–7
