# Supplementary material for: Quantitation of putative colorectal cancer biomarker candidates in serum extracellular vesicles by targeted proteomics
Source: Sci Rep. 2017 Oct 6;7:12782. doi: 10.1038/s41598-017-13092-x (PMC5630664; doi:10.1038/s41598-017-13092-x)

**Supplementary Information**

**Quantitation of putative colorectal cancer biomarker candidates in serum extracellular vesicles by targeted proteomics**

*Takashi Shiromizu ^†^, Hideaki Kume^†^, Mimiko Ishida^†^, Jun Adachi^†^, Masayuki Kano‡, Hisahiro Matsubara‡ and Takeshi Tomonaga*^,†^*

^†^Laboratory of Proteome Research, National Institute of Biomedical Innovation Health and Nutrition, Osaka, Japan

*‡Department of Frontier Surgery, Graduate School of Medicine, Chiba University, Chiba, Japan.*

**Author information**

**Affiliations**

**Laboratory of Proteome Research, National Institute of Biomedical Innovation, Health, and Nutrition, 7-6-8 Saito-Asagi, Ibaraki, Osaka 567-0085, Japan**

Takashi Shiromizu, Hideaki Kume, Mimiko Ishida, Jun Adachi, and Takeshi Tomonaga

**Department of Frontier Surgery, Graduate School of Medicine, Chiba University, 1-8-1, Inohana, Chuo-ku, Chiba-shi, Chiba, 260-8670 Japan.**

Masayuki Kano, Hisahiro Matsubara

*** Corresponding author:** Takeshi Tomonaga

**Figure legends**

Supplementary Figure 1: Statistical analysis of target peptides. Relative quantitation of peptides between three cohorts by SRM analysis (N; healthy control, C; CRC without metastasis, Cm; CRC with metastasis). Graph of dot plot indicate the peak area ratio of the endogenous peptide to that of the SI-peptide. (*; p<0.05, **; p<0.01, N.S; not significant)

Supplementary Figure 2: ROC curve analysis for discriminating N and C (red line) or C and Cm (blue line). The area under the curve (AUC) for the discrimination is shown on each graph.

Supplementary Figure 3: ROC curve analysis for combination of target peptides. The diagnostic sensitivity of peptide combination was evaluated between N and C. The area under the curve (AUC), sensitivity and specificity　were shown on each graph

Supplementary Figure 4: Comparison of sensitivity of target peptides with CEA. Sensitivity is calculated as the percentage of sample when cut-off value is set at the maximum peak area of N (red dashed line; specificity=100%).

Supplementary Figure 5: Statistical analysis of target peptides in another cohort. Relative quantitation of peptides among three groups using SRM analysis (N; healthy control, C1; CRC stage 1, C2; CRC stage 2). Graph of dot plot indicates the peak area ratio of the endogenous peptide to that of the SI-peptide. (*; p<0.05, **; p<0.01, N.S; not significant)

Supplementary Figure 6: ROC curve analysis for discriminating N and C1 (green line) or C and C2 (red line) in another cohort. The area under curve (AUC) for the discrimination is shown on each graph.

Supplementary Figure 7: Comparison of sensitivities and specificities of target peptides with CEA in another cohort. Sensitivity and specificity are calculated as the percentage of sample using cut-off values obtained by the training study.

Supplementary Figure 1


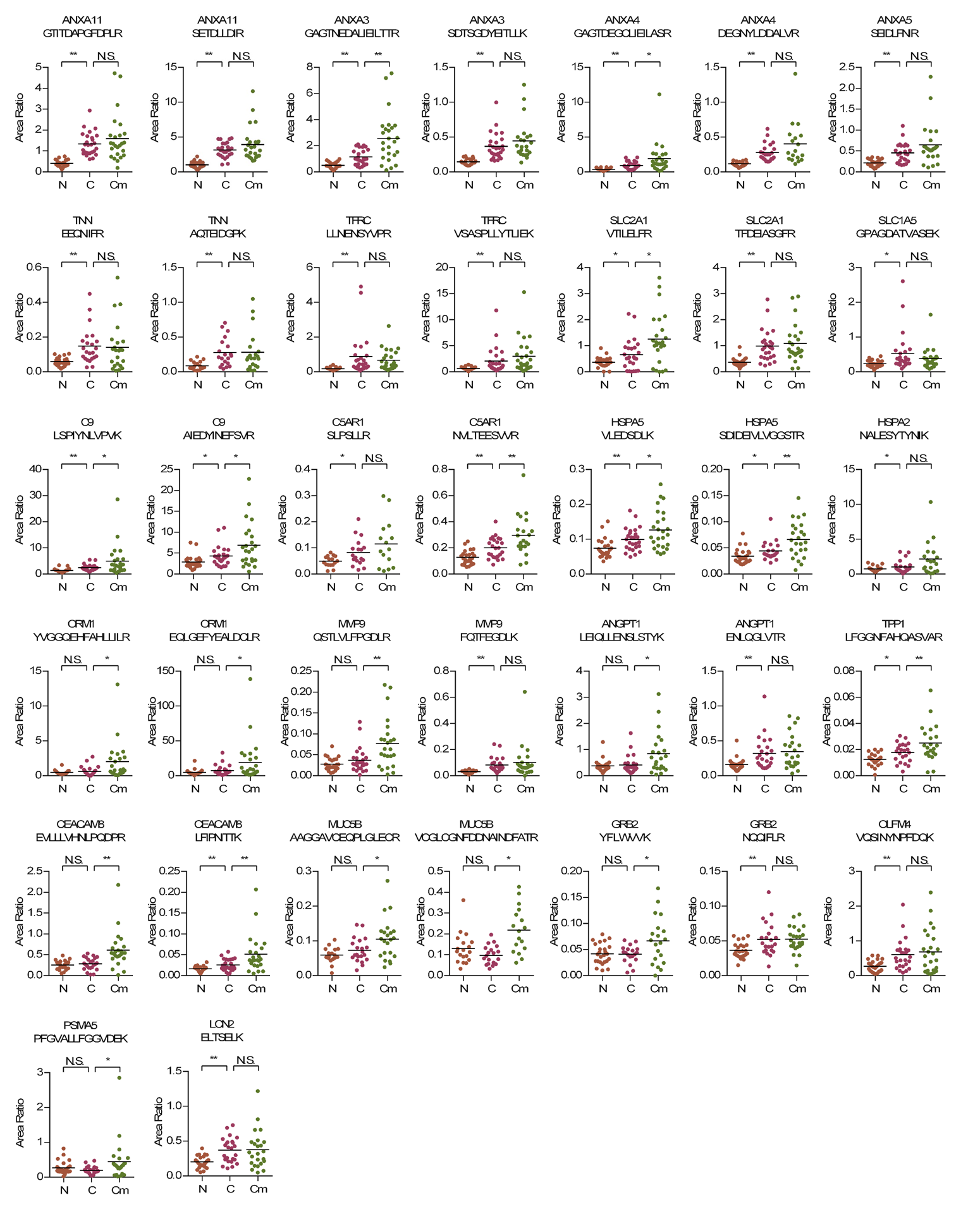


Supplementary Figure 2


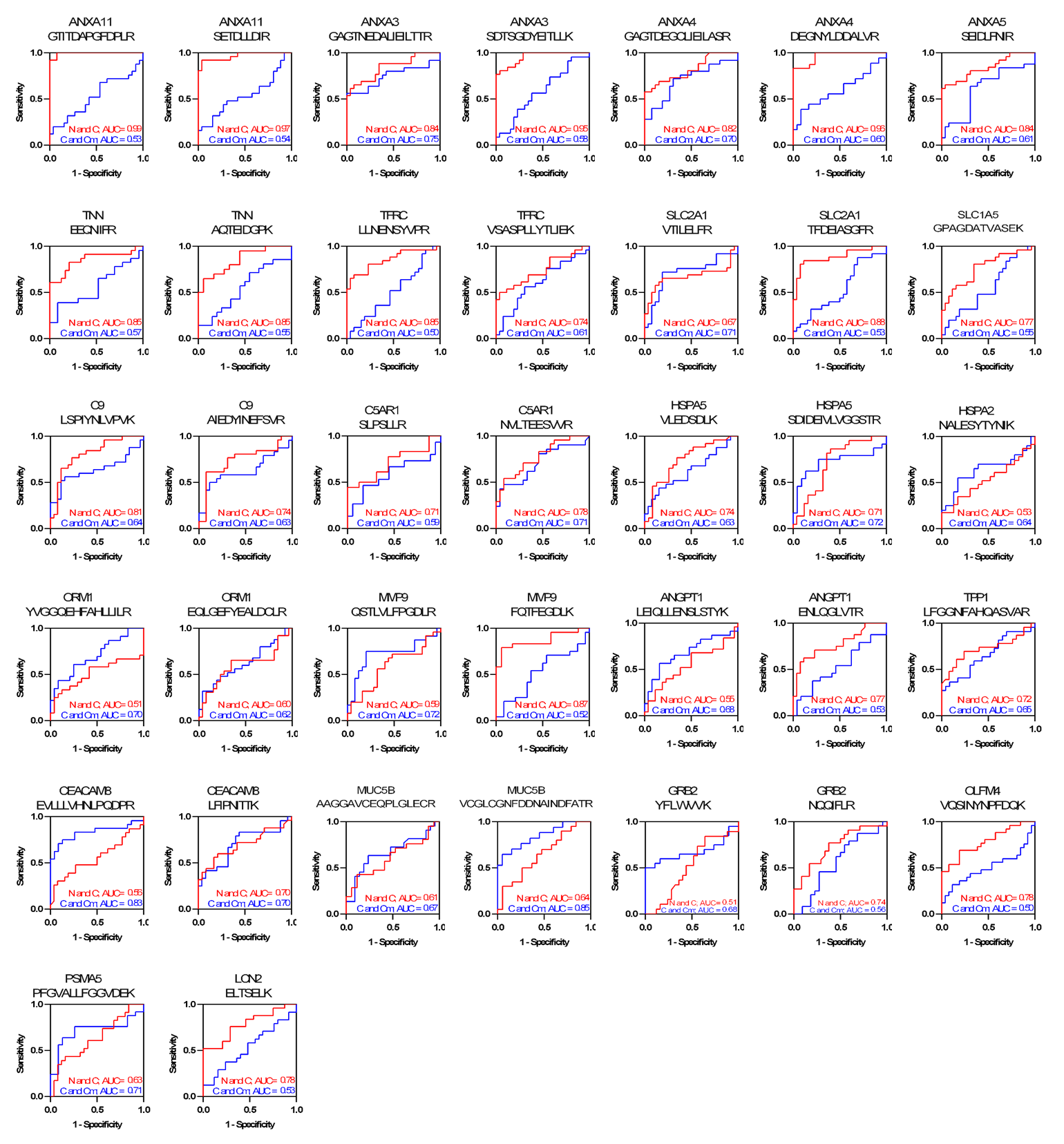


Supplementary Figure 3


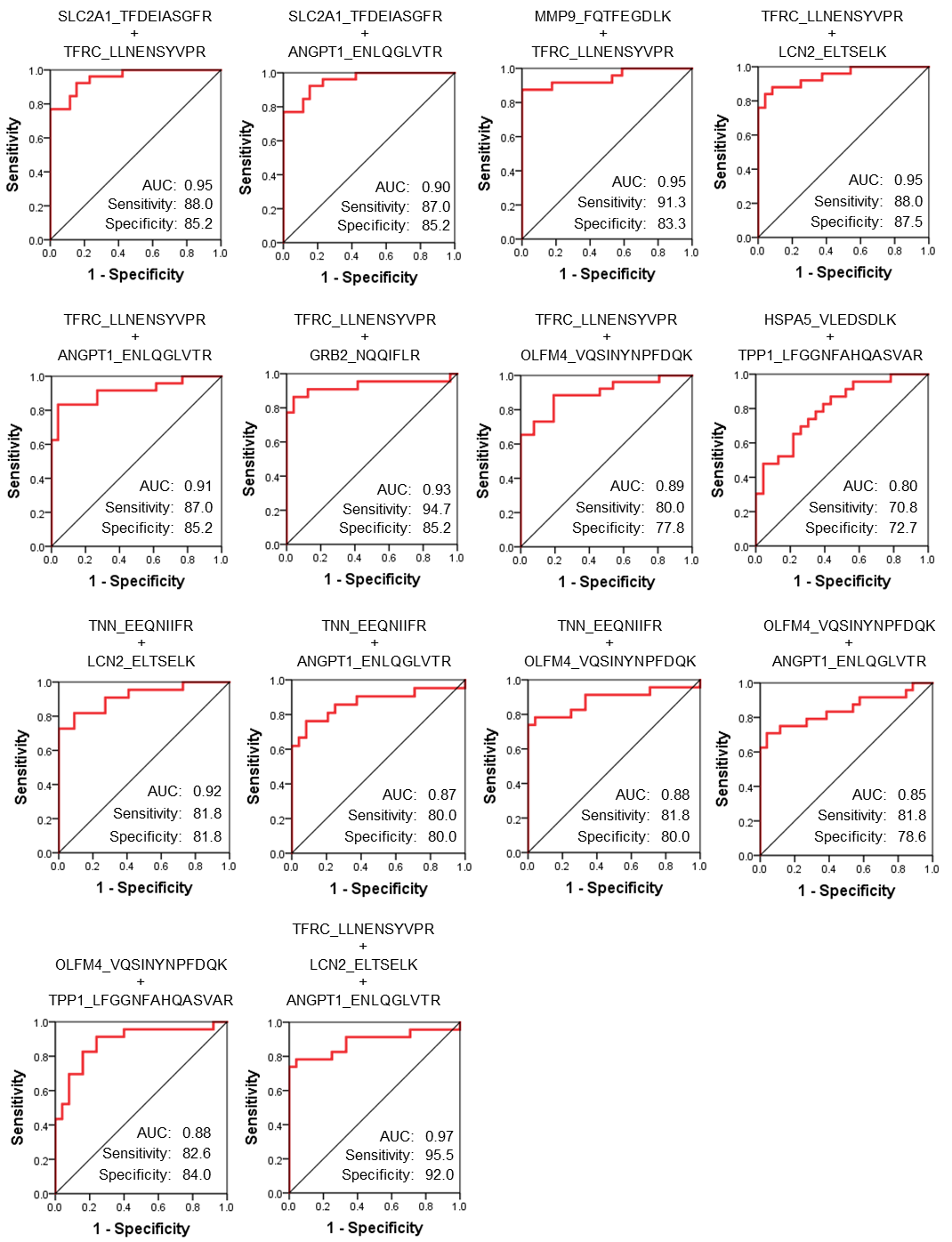


Supplementary Figure 4


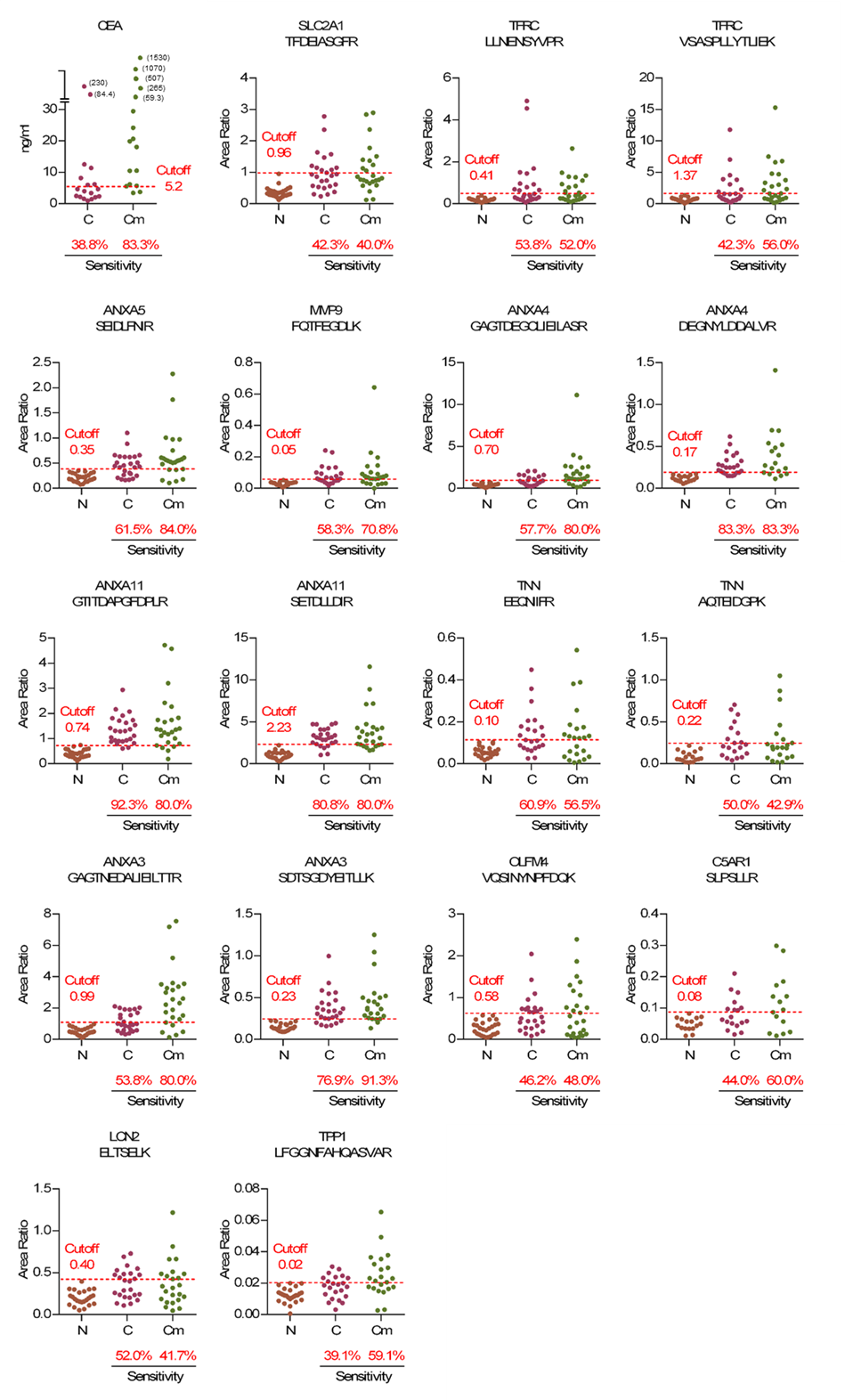


Supplementary Figure 5


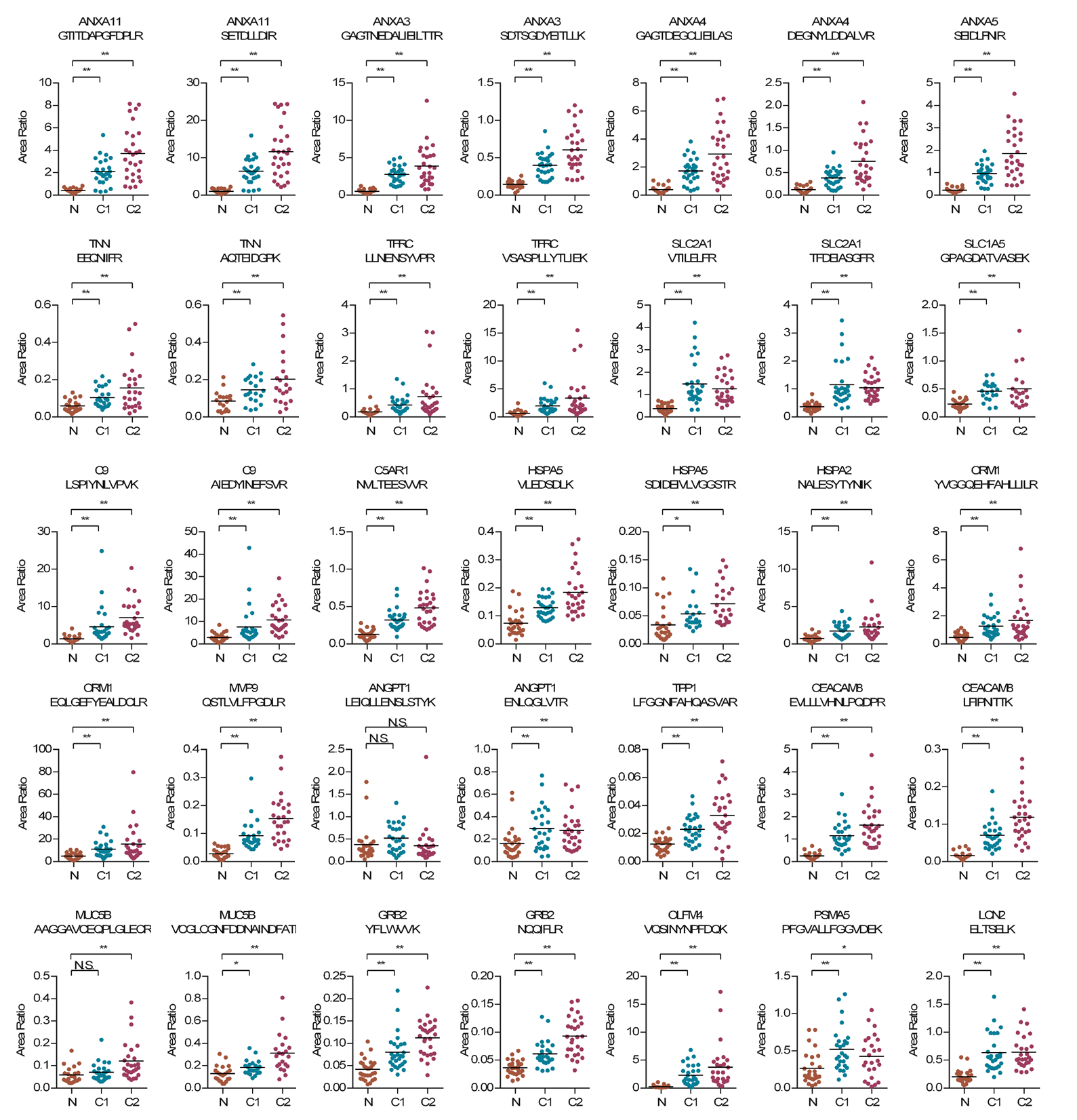


Supplementary Figure 6


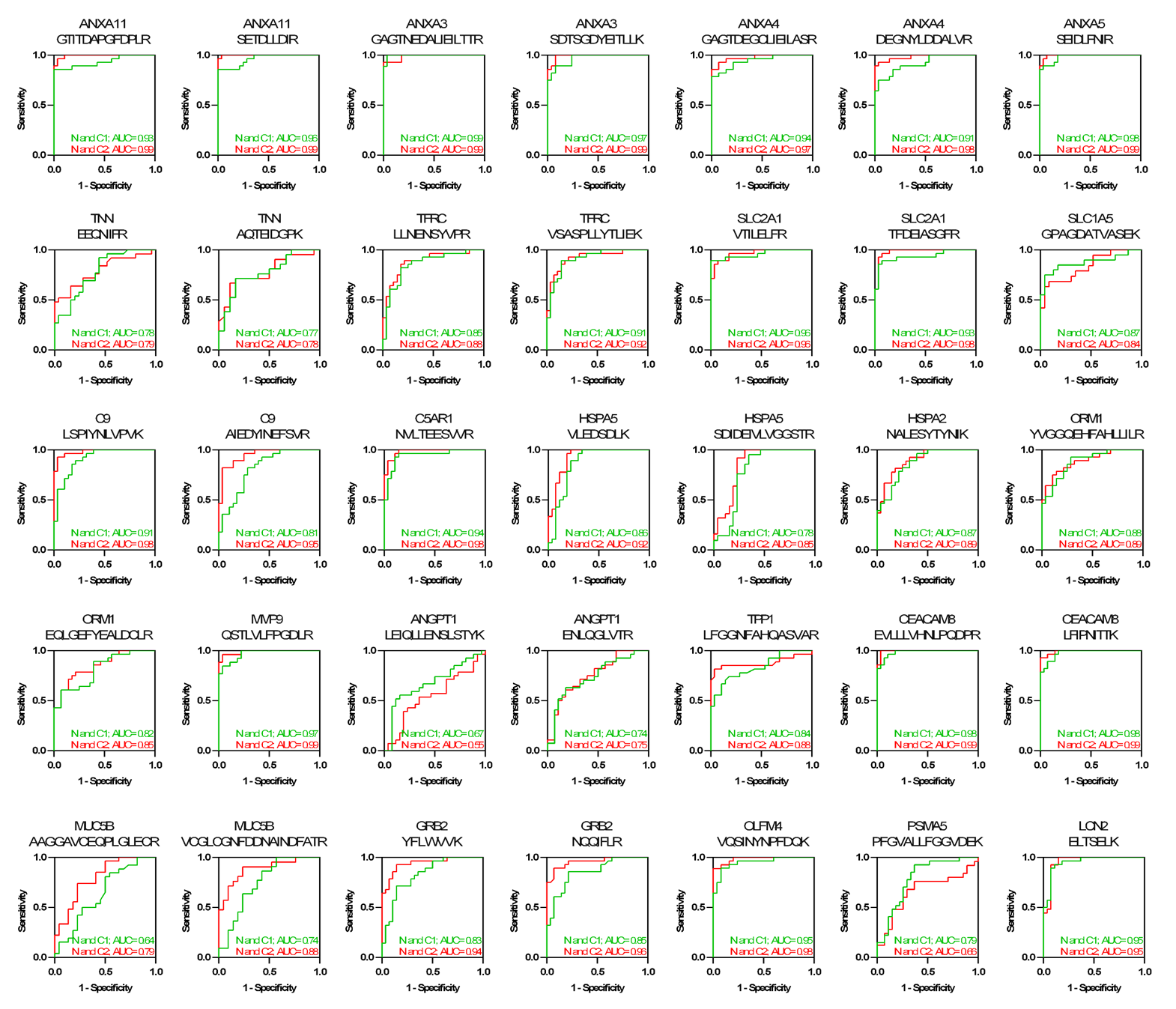


Supplementary Figure 7


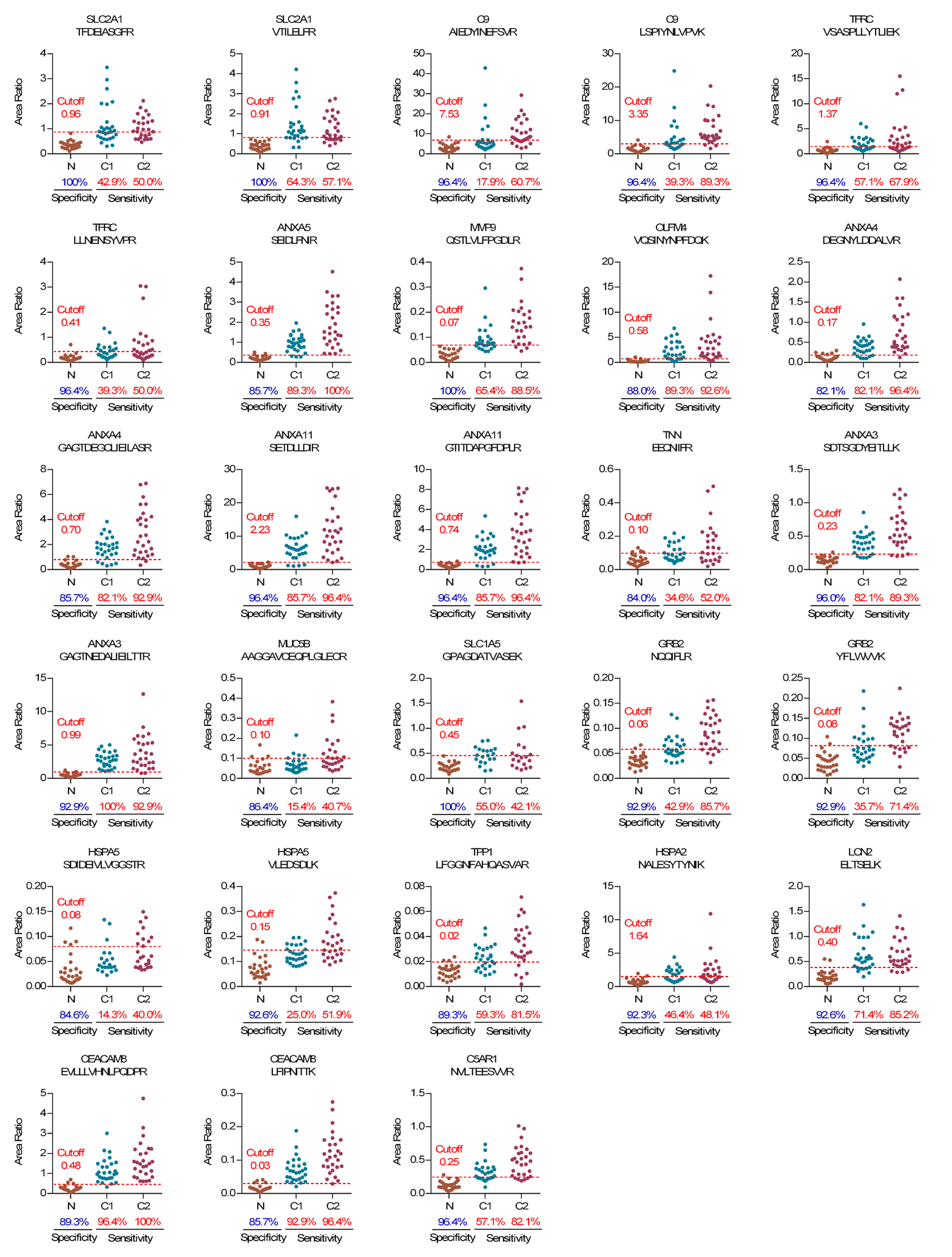

Supplement: Supplementary file 1 — Supplementary Figure 1–7 [file 41598_2017_13092_MOESM1_ESM.doc]
